# The Use of Strontium-90 Beta Radiotherapy as Adjuvant Treatment for Conjunctival Melanoma

**DOI:** 10.1155/2013/349162

**Published:** 2013-01-31

**Authors:** Victoria M. L. Cohen, Vasilios P. Papastefanou, S. Liu, Ian Stoker, John L. Hungerford

**Affiliations:** ^1^Ocular Oncology Service, St Bartholomew's Hospital and Moorfields Eye Hospital, West Smithfield, London EC1A 7BE, UK; ^2^Radiotherapy Department, St Bartholomew's Hospital, London EC1A 7BE, UK

## Abstract

*Background/Aims*. To report the safety and efficacy of strontium (Sr^90^) beta radiotherapy as adjuvant treatment for conjunctival melanoma. *Methods*. A retrospective cohort study was undertaken from 1999 to 2007 of all patients who underwent Sr^90^ beta radiotherapy for incompletely excised conjunctival melanoma. Failure of treatment was defined as recurrence of a conjunctival melanoma at the same location following beta radiotherapy. *Results*. Twenty patients underwent Sr^90^ beta radiotherapy for incompletely excised conjunctival melanoma. Median follow-up interval was 59 months (8–152). All patients had conjunctival melanoma involving the bulbar conjunctiva. Underlying diagnoses included PAM with atypia in 60% (12 of 20), PAM without atypia in 15% (3 of 20), and de novo conjunctival melanoma in 25% (5 of 20). Following Sr^90^ beta radiotherapy, in 90% (18 out of 20) local control was achieved and visual acuity was not affected in any patient. Three patients (15%) had dry eye symptoms, episcleritis, and descemetcoele, respectively. No cataract or secondary glaucoma was reported. *Conclusions*. Sr^90^ treatment is a very effective adjuvant treatment after excisional biopsy and cryotherapy for conjunctival melanoma with a local success rate of 90%. The treatment is not associated with significant side effects and visual acuity is not affected.

## 1. Introduction

Conjunctival melanoma accounts for 1-2% of all ocular melanomas [1]. The management of conjunctival melanoma is fraught with difficulties. The majority of patients have the associated condition of primary acquired melanosis (PAM) with atypia and are therefore prone to multiple new tumours throughout their lifetime [2, 3]. Recurrent or incompletely excised conjunctival melanoma is associated with an increased risk of metastases [3]. Therefore, primary conjunctival melanoma is managed by complete excision and double freeze-thaw cryotherapy. Adjuvant therapy is necessary to improve local tumour control and survival of the patient especially when the histopathology reports indicate tumour is present at the surgical margins. Adjuvant therapy includes cryotherapy [4], topical mitomycin C [5], brachytherapy [6–8], proton beam radiotherapy [9], or rarely alpha 2b interferon [10]. Brachytherapy includes the use of ruthenium-106, [6, 7] iodine-191 [8], or strontium-90 beta radiotherapy. 

**Figure 1:**
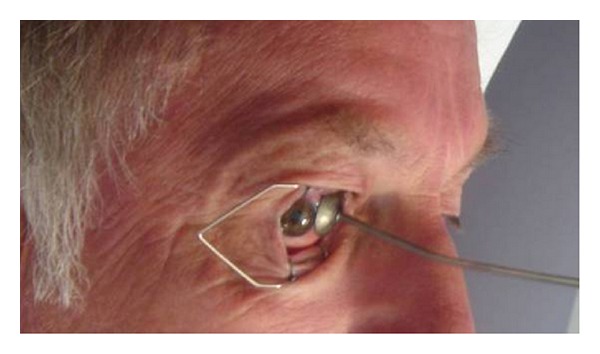
Placement of strontium ophthalmic applicator in the nasal bulbar conjunctiva as adjuvant treatment for conjunctival melanoma.

**Figure 2:**
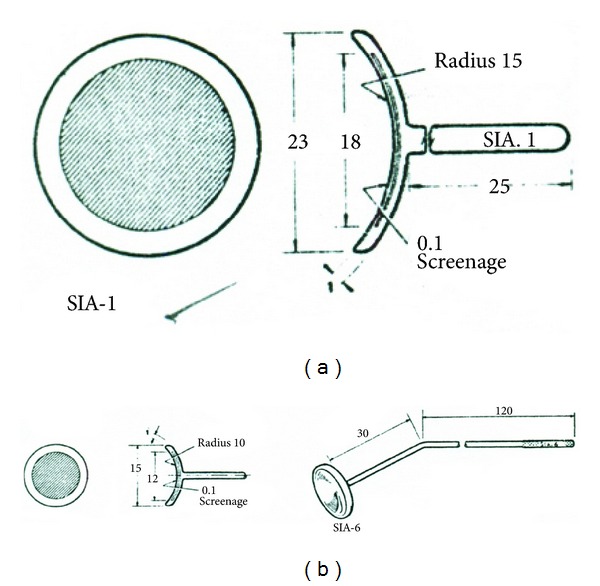
Strontium-90 ophthalmic applicators used. (a) SIA-1 ophthalmic applicator. (b) SIA-6 ophthalmic applicator. Note the central active area with a peripheral inactive rim (see Section 2).

Strontium-90 (Sr^90^) beta radiotherapy is a noninvasive treatment using a hand held applicator (Figure 1). It has also been used for controlling wound healing after glaucoma drainage surgery [11]. It is only available for the treatment of ocular surface tumours in the London Ocular Oncology Service: the other two ocular oncology centres in the UK do not own an applicator as they are no longer produced by Bebig. It is administered under topical anaesthetic in the outpatient setting. Treatments are fractionated to minimise radiation complications. The Sr^90^ ophthalmic applicators [Bebig] (Figure 2) consist of a radioactive source and a shielded holder for the source. Radioactive source consists of a palladium coating, an encapsulating material of silver and strontium-90 is the encapsulated material. Sr^90^ is a radioactive substance with a long half-life of 28.6 years and a maximum energy of 2.3 MeV. It decays to Yttrium-90 with a half-life of 29 years with the emission of a beta particle of maximum energy of 0.55 MeV. Yttrium-90 decays to stable zirconium-90 with emission of a beta particle of minimum energy of 2.27 MeV [12]. 

The holder of the source consists of a shielding plate made from polymethylmethacrylate (PMMA), a fixation ring for the shielding plate and a holder with mechanism to grab the source. Two applicators of different size were used depending on the area of the lesion. SIA-1 applicator has an active disc of 18 mm and an activity of 10 mCi with an approximate surface/dose rate of 2 rads/sec. SIA-6 applicator has an active disc of 12 mm and an activity of 10 mCi with an approximate surface/dose rate of 4 rads/sec. These applicators have an outer peripheral rim of about 2 mm that is inactive (Figure 2).

The data available for the usage of Sr^90^ as an adjuvant treatment in conjunctival melanoma is limited. The aim of this study is to examine the efficacy and safety of Sr^90^ applicators as adjuvant treatment in a cohort of patients with conjunctival melanoma. 

## 2. Materials and Methods

A retrospective cohort study was undertaken from 1999 to 2011 of all patients who underwent Sr^90^ beta radiotherapy for incompletely excised conjunctival melanoma. The parameters assessed were the patients' age, sex and predisposing diagnosis, pre- and posttreatment visual acuity, applicator type, and length of followup and tumour parameters including location, size, and histopathology results. All patients were treated with five fractions of 10 Gy hence the total dose was 50 Gy administered to the scleral surface. Because the type of radiation used is not very penetrating, the dose falls of very rapidly with depth and is only around half this by the time it has reached 1 mm into the tissue.

The main outcomes measured were melanoma recurrence at the treatment site and ocular complications. Failure of treatment was defined as the development of recurrence in the treated area. Development of melanoma in other nontreated areas was not characterized as failure of treatment as many patients with conjunctival melanoma have PAM with atypia.

All data were entered in an Excel database and statistical analysis was performed with the use of SPSS 13.0. Kaplan-Meier analysis was performed to determine the recurrence free disease at treatment site at 5 and 10 years.

## 3. Results 

Over 8 years, twenty patients (11 males and 9 females) with biopsy proven conjunctival melanoma were treated with adjuvant Sr^90^ beta radiotherapy. Median age of the patients was 68 yrs (range 54–92 yrs). Median followup was 59 months (8–152 months).  Table 1 presents patients' clinical data. 

In regards to predisposing conjunctival pathology, eleven patients had associated PAM with atypia, (12/20, 60%), three patients had associated PAM without atypia (3/20, 15%), and in five patients conjunctival melanoma developed de novo without the presence of associated PAM (5/20, 25%). 

All patients underwent complete excision of the lesion with adjuvant double freeze-thaw cryotherapy at the margins of the lesion after initial referral. Seventeen patients were treated with the SIA-6 applicator (17/20, 85%) with a median duration of treatment at 236 seconds, 10 Gy per fraction and three patients were treated with the SIA-1 applicator (3/20, 15%) with a median duration of treatment at 767 seconds, 10 Gy per fraction. The total number of fractions was always five.

In all cases Sr^90^ beta radiotherapy was applied as adjuvant treatment. In seventeen cases (17/20, 85%) Sr^90^ radiotherapy was applied as adjuvant treatment following excisional biopsy and cryotherapy of a new conjunctival melanoma. The interval between primary treatment and Sr^90^ radiotherapy ranged from 1 to 12 months with a mean of 3 months. In three cases (3/20, 15%) treatment was applied as adjuvant treatment of a recurrent conjunctival melanoma following excision and cryotherapy. Two of the three recurrent cases had failed treatment with excision biopsy and one had failed treatment with topical mitomycin C. None of the patients had received prior adjuvant strontium radiotherapy.

In eleven patients (11/20, 55%) the tumour was located at the temporal bulbar conjunctiva, in three patients inferotemporally (3/20, 15%) and in the remaining seven patients in other locations (Table 1). Most of the cases presented with limbal involvement (15/20, 75%). In 9 out of 18 patients (50%) tumour thickness was more than 1 mm.

**Figure 3:**
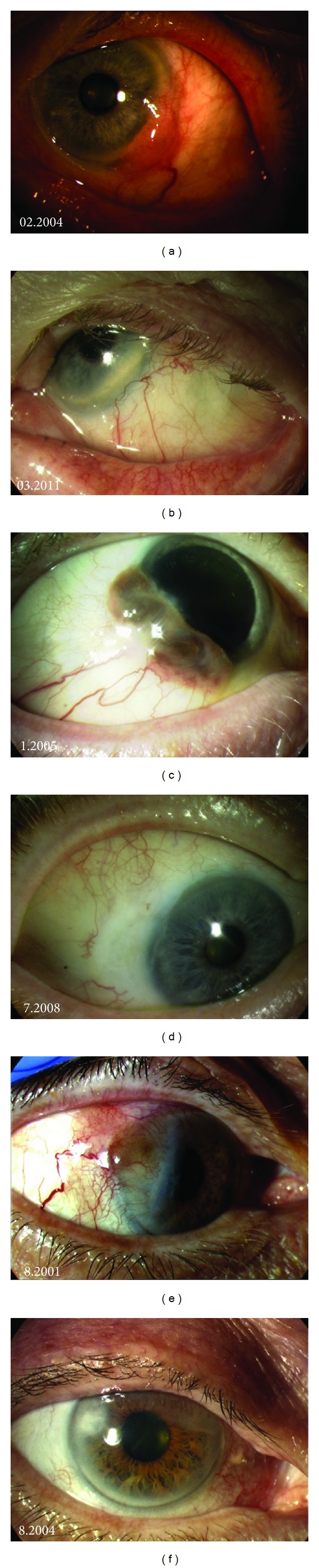
(a) An 86-year-old patient with a de novo temporal conjunctival melanoma that was treated with excisional biopsy and cryotherapy and adjuvant strontium radiotherapy. (b) Seven years later, note the surgical corneal scar but no evidence of recurrence in the treated area. (c) A 69-year-old patient with a predisposing diagnosis of PAM with atypia developed an inferotemporal conjunctival melanoma that was subjected to excisional biopsy and cryotherapy and adjuvant strontium radiotherapy (d) 42 months later, no evidence of disease in the treated area. (e) A 72-year-old patient with a predisposing diagnosis of PAM with atypia developed a temporal conjunctival melanoma adjacent to the limbus that was treated with excisional biopsy and cryotherapy and adjuvant strontium radiotherapy. (f) Three years later, note the surgical corneal scar but no evidence of disease in the treated area.

**Figure 4:**
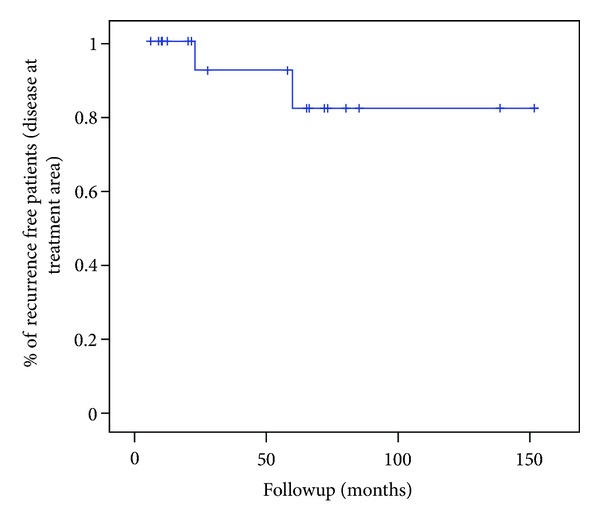
Kaplan-Meier analysis plot. Estimated percentage of recurrence free patients with strontium treatment was 82% at 5 and 10 years.

Eighteen out of 20 patients (90%) were tumour-free in the treated area at the end of follow-up period (Figure 3). Estimated percentage of recurrence free disease was 82% at 5 and 10 years (Figure 4). Only two patients (2/20, 10%) developed a recurrence in the treated area at 15 months and 37 months after treatment, respectively. Area of recurrence was treated with lamellar scleral dissection and adjuvant cryotherapy in one case and excisional biopsy and cryotherapy in the other.

Following adjuvant Sr^90^ beta radiotherapy, 85% of patients (17/20) suffered no ocular complications. There was no difference on the pre- and post-treatment visual acuity in any patient (see Table 2). This is an important result as it illustrates that no visually significant cataract developed during the period of followup which ranged up to 12 years. One patient had transient episcleritis that responded to local anti-inflammatory treatment (1/20, 5%), one had transient dry eye symptoms (1/20, 5%) and there was another single case of a descemetocele (1/20, 5%).

## 4. Discussion

The use of Sr^90^ applicators for the treatment of conjunctival melanoma has not been extensively reported [13–17]. In our study all patients had an initial excisional biopsy followed by cryotherapy and received adjunctive Sr^90^ beta radiotherapy. In other studies Sr^90^ has been either used as primary treatment or as adjuvant treatment with or without cryotherapy [13–17].

A total dose of 50 Gy was administered. From other studies (Table 3) there does not appear to be a standardized regimen but on average, the total dose is 36–60 Gy and fraction size is typically 10 Gy.

Success rate at the end of follow-up period was 90%. A previous study showed a success rate of 80% for a total dose of 54 Gy [15]. A recent case series had a success rate of 95% for a total dose of 60 Gy and 43% for a total dose of 35 Gy highlighting that delivery of less than 40 Gy in the treated area is probably not sufficient for tumour control [16].

Local complications were not severe. One patient developed episcleritis and one patient developed a descemetocele. None of our patients developed neovascular glaucoma or cataract. Visual acuity was not affected in any of our patients at the end of follow-up period. Scleral melting and transient dry eye symptoms have been previously reported [16]. Scleral melting was not noted in this series. Primary Sr^90^ treatment for conjunctival melanoma has been associated with telangiectasia, discomfort, and cataract [13, 14]. No such complications were noted with adjuvant Sr^90^ radiotherapy.

Sr^90^ radiotherapy appears to be safer and more effective in comparison to other proposed methods of adjuvant treatment for conjunctival melanoma. In comparison to topical mitomycin C, no patient experienced discomfort or pain after treatment and none of the long-term complications such as punctal stenosis, limbal stem cell deficiency, or keratoconjunctivitis were noted [5, 18, 19]. Reported recurrence rates for topical mitomycin C from various case series were 20–50% [5, 18–20]. In our study recurrence rate following Sr^90^ beta radiotherapy was 10%. Topical Interferon alpha 2b results were encouraging in a small case series with no local complications [10]; however, no long-term data is available. Ruthenium plaque brachytherapy has been used as adjuvant treatment with delivery of a high dose of 290–320 Gy [6, 7] or low dose of 100 Gy [1] but these publications are confined to case reports. Proton bean radiotherapy has been used widely as adjuvant treatment in recurrent conjunctival melanoma and is associated with high rates of severe local complications including sicca syndrome, eyelash loss, cataract, limbal stem cell deficiency, and squamous metaplasia of the corneal epithelium and neovascular glaucoma which sometimes necessitates removal of the eye [9, 21].

Despite the excellent therapeutic outcome, Sr^90^ beta radiotherapy has limitations. The location and size of the tumour are of paramount importance in deciding which adjuvant treatment to use. Sr^90^ beta radiotherapy is limited to use on the bulbar conjunctiva. This is due to the shape and size of the applicator. Custom designed iodine plaques that are reverse mounted can be used to treat tarsal conjunctival melanoma following primary excision (communication with Dr. Carol and Jerry Shields). The additional problem of Sr^90^ beta radiotherapy is its availability. The applicators are no longer being produced due to low demand. This is a problem for Ocular Oncologists in new centres and Glaucoma specialists, especially those practicing in developing countries. Thankfully, due to the long half-life of Strontium, most applicators when cared for correctly are still in use even 20–30 years on.

In conclusion, the present study demonstrates that adjuvant Sr^90^ beta radiotherapy is safe and effective at preventing local recurrence of conjunctival melanoma.

## Figures and Tables

**Table 1:** Clinical data of participating patients.

Patient	Age	Gender	Tumor location	Tumor size (mm)	Tumor thickness (mm)	TNM Classification^1^	Primary excision and cryotherapy	Mode of strontium treatment	Time to strontium treatment (months)	Type of applicator
1	58	F	Inferior temporal	5 × 3	0.6	T1N0MX	Y	Adjuvant (base and lateral)	3	SIA 6
2	86	F	Temporal	8 × 4	1	T2N0MX	Y	Adjuvant (base and lateral)	2	SIA 6
3	72	M	Inferior temporal	5 × 5	1	T4N0MX	Y	Base adjuvant	3	SIA 6
4	71	M	Inferior nasal	10 × 7	6	T1N0MX	Y	Adjuvant (base and lateral)	3	SIA 6
5	92	F	Temporal	6 × 4	2.5	T2N0MX	Y	Adjuvant (base and lateral)	3	SIA 6
6	72	M	Temporal	0.7 × 0.3	Not assessed thoroughly	T4N0MX	Y	Secondary to recurrence	4	SIA 6
7	63	F	Temporal	6 × 4	0.6 mm	T2N0MX	Y	Adjuvant (base and lateral)	2	SIA 6
8	69	M	Superior nasal	3 × 1.8	1.9	T1N0MX	Y	Adjuvant (base and lateral)	3	SIA 6
9	61	F	Temporal	2 × 2	4	T1N0MX	Y	Adjuvant (base and lateral)	3	SIA 6
10	68	F	Temporal	9 × 3	1 mm	T2N0MX	Y	Secondary to recurrence	4	SIA 6
11	68	M	Temporal	14 × 8	3	T2N0MX	Y	Adjuvant (base and lateral)	1	SIA 1
12	76	M	Inferior temporal	11 × 4.5	6	T2N0MX	Y	Adjuvant (base and lateral)	2	SIA 6
13	65	F	Temporal	10 × 8	1.3	T2N0MX	Y	Adjuvant (base and lateral)	3	SIA 6
14	60	M	Nasal	7 × 5	2	T2N0MX	Y	Secondary to recurrence	3	SIA 1
15	69	M	Temporal	13 × 6	1.5	T2N0MX	Y	Adjuvant (base and lateral)	1	SIA 1
16	67	M	Superonasal	6 × 1	1	T1N0MX	Y	Secondary to recurrence	1	SIA 6
17	63	F	Temporal	7 × 2.5	1	T2N0MX	Y	Adjuvant (base and lateral)	2	SIA 6
18	54	F	Temporal	6 × 3	1	T2N0MX	Y	Adjuvant (base and lateral)	1	SIA 6
19	69	F	Superior	N/A	N/A	T1N0MX	Y	Adjuvant (base and lateral)	2	SIA 6
20	72	M	Superior	1.5 × 1.0	Not assessed thoroughly	T1N0MX	Y	Adjuvant (base and lateral)	4	SIA 6

^1^TNM classification primary tumour (T): TX: primary tumour cannot be assessed, T0: no evidence of primary tumour, T1: tumour of bulbar conjunctival occupying one quadrant or less, T2: tumour of the bulbar conjunctiva occupying more than 1 quadrant, T3: tumour of conjunctival fornix and palpebral conjunctiva or caruncle, T4: tumour invades eyelid, cornea, or orbit. Regional lymph nodes (N): N: NX: regional lymph nodes cannot be assessed, N0: no regional lymph node metastasis, N1: regional lymph node metastasis. Distant metastasis (M): MX: distant metastasis cannot be assessed, M0: no distant metastasis, M1: distant metastasis.

*SIA 6: 12 mm ophthalmic applicator, SIA 1: 18 mm ophthalmic applicator.

**Table 2:** Visual acuity in patients with conjunctival melanoma receiving adjunctive Sr^90^ treatment.

Patient	Visual acuity before TX	Visual acuity after TX
1	6/5	6/5
2	6/9	6/9
3	6/6	6/9
4	6/6	6/6
5	6/9	6/9
6	6/18	6/18
7	6/9	6/9
8	6/5	6/6
9	6/6	6/6
10	6/6	6/6
11	6/6	6/6
12	6/9	6/9
13	6/9	6/9
14	6/5	6/5
15	6/6	6/5
16	6/6	6/5
17	6/6	6/6
18	6/12	6/9
19	6/5	6/6
20	6/18	6/18

**Table 3:** Comparison of studies examining Sr^90^ radiotherapy in the treatment of conjunctival melanoma.

							Local complications							
Author	Pts	Primary TX	Adjuvant TX	Dosimetry (total)	Followup	Success rate (%)	Scleral melting	Dry eye	Descemetocele	Episcleritis	Telangiectasia	Cataract	Secondary glaucoma	Corneal opacities
Lommatzsch [13, 14]	45	Sr^90^ beta radiotherapy	None	10 to 200 Gy^1^	Up to 14 years	70% (31/45)	0	0	0	0	18	7	2	3
	20	Excisional biopsy	Sr^90^	10 to 200 Gy^1^	Up to 14 years	Not specified^2^								
Krause et al. [15]	15	Excisional biopsy	Sr^90^	54 Gy	1–5 years	80% (12/15)	Not addressed							
Missotten et al. [16]^3^	46	Excisional biopsy^3^	Sr^90^	60 Gy (*n* = 38) 35.4 Gy (*n* = 7)	8 years mean	95% (36/38) 43% (3/7)	1	Unspecified number^4^	0	0	0	0	0	0
Our study	20	Excisional biopsy with cryotherapy	Sr^90^	50 Gy	0.5–10 years	90% (18/20)	0	1	1	1	0	0	0	0

^1^Unknown number of fractions.

^2^Overall success rate 75% (51/65).

^3^Abstract currently available. It is unclear from the publicly available data whether these patients were subjected to cryotherapy.

^4^Abstract currently available. It is unclear from the publicly available data what is the exact number of patients with dry eye symptoms described as being transient.

## References

[1] Damato B, Coupland SE (2009). An audit of conjunctival melanoma treatment in Liverpool. *Eye*.

[2] Shields CL, Shields JA, Gündüz K (2000). Conjunctival melanoma: risk factors for recurrence, exenteration, metastasis, and death in 150 consecutive patients. *Archives of Ophthalmology*.

[3] Shields CL, Markowitz JS, Belinsky I (2011). Conjunctival melanoma: outcomes based on tumor origin in 382 consecutive cases. *Ophthalmology*.

[4] Jakobiec FA, Rini FJ, Fraunfelder FT, Brownstein S (1988). Cryotherapy for conjunctival primary acquired melanosis and malignant melanoma. Experience with 62 cases. *Ophthalmology*.

[5] Ditta LC, Shildkrot Y, Wilson MW (2011). Outcomes in 15 patients with conjunctivalmelanoma treated with adjuvant topical mitomycin C: complications and recurrences. *Ophthalmology*.

[6] Zehetmayer M, Menapace R, Kulnig W (1993). Combined local excision and brachytherapy with ruthenium-106 in the treatment of epibulbar malignancies. *Ophthalmologica*.

[7] Krause L, Mladenova A, Bechrakis NE (2009). Treatment modalities for conjunctival melanoma. *Klinische Monatsblätter für Augenheilkunde*.

[8] Shields JA, Shields CL, Freire JE, Brady LW, Komarnicky L (2003). Plaque radiotherapy for selected orbital malignancies: preliminary observations—the 2002 montgomery lecture, part 2. *Ophthalmic Plastic and Reconstructive Surgery*.

[9] Wuestemeyer H, Sauerwein W, Meller D (2006). Proton radiotherapy as an alternative to exenteration in the management of extended conjunctival melanoma. *Graefe’s Archive for Clinical and Experimental Ophthalmology*.

[10] Herold TR, Hintschich C (2010). Interferon alpha for the treatment of melanocytic conjunctivallesions. *Graefe's Archive for Clinical and Experimental Ophthalmology*.

[11] Kirwan JF, Constable PH, Murdoch IE, Khaw PT (2003). Beta irradiation: new uses for an old treatment: a review. *Eye*.

[12] Potter R, van Limbergen E (2012). Uveal melanoma 591-610. *GEC ESTRO Handbook of Brachytherapy*.

[13] Lommatzsch PK (1977). Beta irradiation of conjunctival melanomas. *Transactions of the Ophthalmological Societies of the United Kingdom*.

[14] Lommatzsch PK (1978). Beta-Ray treatment of malignant epibulbarmelanoma. *Albrecht von Graefe's Archive for Clinical and Experimental Ophthalmology*.

[15] Krause L, Ritter C, Wachtlin J (2008). Recurrence rate following adjuvant strontium-90 brachytherapy after excision of conjunctivalmelanoma. *Klinische Monatsblätter für Augenheilkunde*.

[16] Missotten G, De Keizer RJW, Spileers W (2011). Strontium brachytherapy in conjunctival melanoma (abstract). *Acta Ophthalmol*.

[17] Missoten G, Tassignon MJ, De Kaizer RJX Strontium brachytherapy in conjunctival melanoma(abstract). SBO-BOG. http://www.ophthalmologia.be/plugins/abstracts/view_abstract.php?abs_id=1402.

[18] Khong JJ, Muecke J (2006). Complications of mitomycin C therapy in 100 eyes with ocular surface neoplasia. *British Journal of Ophthalmology*.

[19] Russell HC, Chadha V, Lockington D, Kemp EG (2010). Topical mitomycin C chemotherapy in the management of ocular surface neoplasia: a 10-year review of treatment outcomes and complications. *British Journal of Ophthalmology*.

[20] Finger PT, Czechonska G, Liarikos S (1998). Topical mitomycin C chemotherapy for conjunctival melanoma and PAM with atypia. *British Journal of Ophthalmology*.

[21] Westekemper H, Anastassiou G, Sauerwein W (2006). Analysis of ocular surface alterations following proton beam radiation in eyes with conjunctival malignant melanoma. *Ophthalmologe*.

